# Mechanistic Insights into Bioengineered Antibiofilm Enamel Pellicles

**DOI:** 10.1177/00220345231162336

**Published:** 2023-04-21

**Authors:** D.G. Moussa, R.W. Kung, J.S. Tse, W.L. Siqueira

**Affiliations:** 1 College of Dentistry, University of Saskatchewan, Saskatoon, Canada; 2 Department of Physics and Engineering Physics, College of Art and Science, University of Saskatchewan, Saskatoon, Canada

**Keywords:** preventive dentistry, salivary proteins and peptides, molecular dynamics simulation, 3D imaging, biofilm, dental caries

## Abstract

Dental caries remains the most widespread chronic disease worldwide. Basically, caries originates within biofilms accumulated on dental enamel. Despite the nonrenewable nature of the enamel tissue, targeted preventive strategies are still very limited. We previously introduced customized multifunctional proteinaceous pellicles (coatings) for controlling bacterial attachment and subsequent biofilm succession. Stemmed from our whole proteome/peptidome analysis of the *in vivo* acquired enamel pellicle, we designed these pellicles using hybrid mixtures of the most abundant and complementary-acting antimicrobial and antifouling proteins/peptides for synergetic suppression of early biofilms. In conjugating these domains synthetically, their bioinhibitory efficacy was remarkably boosted. Herein, we sought to explore the key structure-function relationship of these potent *de novo* hybridized conjugates in comparison with their individual domains, solely or in physical mixtures. Specifically, we interrelated the following facets: physicochemical and 3-dimensional folding characteristics via molecular dynamics simulations, adopted secondary structure by circular dichroism, immobilization capacity on enamel through high–spatial resolution multiphoton microscopy, and biofilm suppression potency. Our data showed consistent associations among the increased preference for protein folding structures, α-helix content, and enamel-immobilization capacity; all were inversely correlated with the attached bioburden. The expressed phenotypes could be explained by the adopted strongly amphipathic helical conformation upon conjugation, mediated by the highly anionic and acidic N-terminal pentapeptide shared region/motif for enhanced immobilization on enamel. In conclusion, conjugating bioactive proteins/peptides is a novel translational approach to engineer robust antibiofilm pellicles for caries prevention. The adopted α-helical conformation is key to enhance the antibiofilm efficacy and immobilization capacity on enamel that are promoted by certain physicochemical properties of the constituent domains. These data are valuable for bioengineering versatile therapeutics to prevent/arrest dental caries, a condition that otherwise requires invasive treatments with substantial health care expenditures.

## Introduction

Dental caries constitutes a major global public health challenge; it is the most common chronic disease and affects >2 billion people (Global Burden of Disease Collaborative Network 2020; World Health Organization 2022). Caries initiates within accumulated dysbiotic biofilms, mostly on the nonrenewable enamel tissue (Selwitz et al. 2007). Consequently, invasive dental treatments have been the typical remediation measure, with their associated massive impacts on health care budgets. For instance, the annual cost of dental care in the United States alone was approximately $136 billion, which represented 3.7% of the country’s total health care expenditures in 2018 (US Department of Health and Human Services 2021).

Although slowly progressing, modern dentistry has been focusing on developing less invasive preventive strategies and implementing programs for caries prevention (US Department of Health and Human Services 2021). Some contemporary approaches, other than dietary and hygienic measures, include probiotics to antagonize accountable species (Lin et al. 2018), targeted antimicrobials to suppress specific pathogens (Guo et al. 2015; Moussa and Aparicio 2020), and/or designed multifunctional constructs to govern biofilm succession (Moussa and Siqueira 2021).

Bioactive proteins/peptides have received remarkable attention as a new category of anticaries therapeutics by promoting hydrophobic and antimicrobial properties (Moussa, Fok, and Aparicio 2019), remineralization potential (Ye et al. 2017), augmented protection against enamel demineralization (Basiri et al. 2017), biofilm dispersion capabilities (Moussa and Siqueira 2021), and others as comprehensively reviewed by Ramburrun et al. (2021). Furthermore, their inherent inability to develop bacterial resistance and their lack of mammalian cell toxicity indicate that these peptides have significant potential as next-generation therapeutics (Fjell et al. 2011).

Our whole proteome/peptidome data of the *in vivo* acquired enamel pellicle showed a distinct abundance of histatins, statherin, and their functional fragments (domains) (Siqueira et al. 2007; Siqueira et al. 2010). While these domains are products of proteins after bacterial cleavage, they inherently maintain their parent function with extra resistance to degradation (Siqueira and Oppenheim 2009). Given the inspiring lifetime enamel-protecting role of the acquired enamel pellicle (Siqueira et al. 2012), we previously presented novel protein/peptide pellicles composed of a mix of synergically acting antimicrobial and antifouling functional domains (statherin and histatin derived, respectively) for efficient biofilm dispersion (Moussa and Siqueira 2021). By conjugating these domains synthetically, the biofilm suppression effect was remarkably boosted and sustained for several days (Moussa and Siqueira 2022).

Herewith, we investigated the central structure-function relationships resulting in antibiofilm potency for these *de novo* designed hybridized conjugates as compared with their constituent domains individually or mixed. We explored the 3-dimensional (3D) structural configurations utilizing molecular dynamics simulations and measured the secondary structural elements using circular dichroism spectroscopy. Furthermore, we assessed the *ex vivo* immobilization capacity of these designed pellicles on human enamel by employing high–spatial resolution multiphoton microscopy for optical sectioning. Understanding these structure-function intercorrelations will open avenues for designing robust bioactive protein/peptide candidates with versatile application potential to prevent dental caries and other oral diseases.

## Materials and Methods

Further details are provided in the Appendix Materials and Methods.

### Ethics Approval and Human Participants

All experimental protocols were approved by the Research Human Ethics Board of the University of Saskatchewan (review 1597), and all methods were carried out in accordance with relevant guidelines and regulations. Participants provided informed consent; after which, stimulated whole saliva samples were collected.

### Proteins/Peptides Synthesis

**Figure 1.**
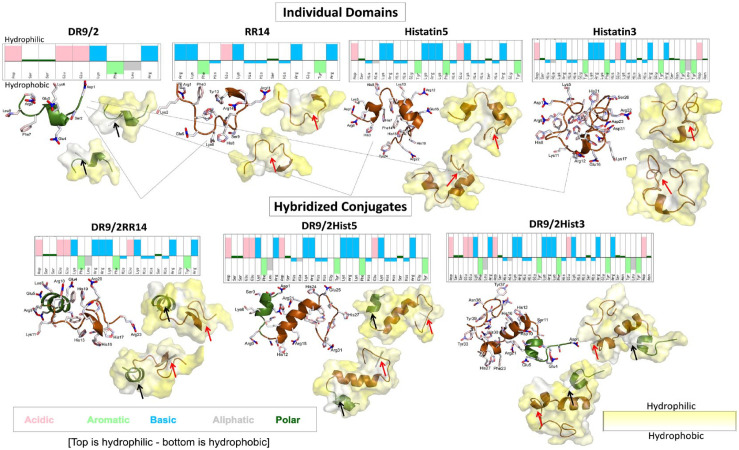
Molecular dynamics simulations for acquired enamel pellicle–derived individual peptide/protein domains and their hybridized conjugates. For each candidate, the sequence is shown with color-coded physicochemical properties of each amino acid residue obtained from https://pepcalc.com/. All the top residues are hydrophilic and the bottom residues are hydrophobic, which are portrayed in the 2-sided hydropathy surface display (yellow, hydrophilic; white, hydrophobic). The 2-sided hydropathy yellow surface plots are taken from opposite sides of each candidate showing the hydrophobic moment vectors. The hydrophobic moment vectors are presented as light gray darts denoted by black and red arrows for the DR9/2 and RR14 regions, respectively. The candidate representative structure is displayed as a bundle of ribbons with side chains shown as sticks, presented on left. The green ribbons refers to the statherin protein family and its functional domain fragment (DR9/2), whereas the brown ribbons refer to the histatins family and its functional domain fragment (RR14) (Moussa and Siqueira 2021). The ribbon bundle representative structure and the top hydropathy surface display are shown in the same orientations.

All protein/peptide candidates were synthesized (purity > 95%) by SynPeptide (Pudong). Their names, sequences, physicochemical, and molecular properties are presented in the Table and Figure 1. A green fluorescent tag (FAM; BOC Sciences) was used to label all tested candidates at the C-termini.

**Table.** Tested Proteins/Peptides Sequences and Their Molecular Properties.

				Net Charge			Charged Residues^a^			HM Vector Length,^b^ kTA°/e	
Protein / Peptide Name: Sequence	MW, g/mol	No. of AA	pI^c^	pH 7	pH 5	GRAVY Index^d^	–	+	Aliphatic Index^e^	DR9/2	RR14
DR9/2^f^											
DSSEEKFLR	1,110.19	9	4.68	–1.1	–0.4	–1.54	3	2	43.33	6.97	NA
RR14^g^											
RKFHEKHHSHRGYR	1,875.06	14	11.00	4.9	8.1	–2.66	1	5	0.00	NA	4.58
Histatin5 (Hist5)											
DSHAKRHHGYKRKFHEKHHSHRGY	3,036.33	24	10.28	6.6	12.2	–2.45	2	7	4.17	NA	5.43
Histatin3 (Hist3)											
DSHAKRHHGYKRKFHEKHHSHRGYRSNYLYDN	4,062.35	32	9.99	6.6	12.4	–2.297	3	8	15.31	NA	5.91
DR9/2RR14^h^											
DSSEEKFLRRKFHEKHHSHRGYR	2,967.26	23	9.99	3.9	7.7	–2.23	4	7	16.96	6.57	8.19
DR9/2Hist5^h^											
DSSEEKFLRDSHAKRHHGYKRKFHEKHHSHRGY	4,128.51	33	9.87	5.6	11.8	–2.21	5	9	14.85	5.65	8.57
DR9/2Hist3^h^											
DSSEEKFLRDSHAKRHHGYKRKFHEKHHSHRGYRSNYLYDN	5,154.58	41	9.70	5.6	12.0	–2.13	6	10	21.46	9.74	9.73

Molecular properties obtained from http://protcalc.sourceforge.net/ for charges and https://web.expasy.org/protparam/ for the rest of the properties.

AA, amino acids; GRAVY, grand average of hydropathy; HM, hydrophobic moment; MW, molecular weight; NA, not applicable; pI, isoelectrical point.

a The – and + signs show the total number of negatively and positively charged residues, respectively.

b HM was calculated as a vector for the DR9/2 and RR14 regions by using the 3D-HM web server (https://www.ibg.kit.edu/HM/).

c pI is the pH at which the molecule carries no net electrical charge.

d The GRAVY value for protein/peptide is calculated as the sum of hydropathy values/hydrophobicity of all the amino acids, divided by the number of residues in the sequence.

e Aliphatic index is the relative volume of a protein occupied by its aliphatic side chains (alanine, valine, isoleucine, and leucine), which are also hydrophobic in nature.

f The DR9/2 peptide is the N-terminus 9–amino acid functional domain of the statherin protein, lacking the covalently linked phosphates at the second and third serine residues.

g The RR14 peptide is the 14–amino acid functional domain derived from the parent Hist3 protein.

h Hybridized conjugate of individual domains.

### Tested Protein/Peptide Groups

Twelve groups were tested: 4 individual protein/peptide domains, 3 hybrid mixtures of dual individual domains, 3 synthetically linked dual hybridized conjugates, pooled filtered whole saliva (positive control), and peptide-free vehicle (0.01% acetic acid; negative control). The individual domains represent the abundant proteins, peptides, and fragments identified in the *in vivo* acquired enamel pellicle (Siqueira et al. 2007; Siqueira and Oppenheim 2009) and include DR9/2, RR14, Histatin5 (Hist5), and Histatin3 (Hist3). DR9/2 is unphosphorylated version of DR9, the N-terminus 9–amino acid functional domain of the statherin protein, lacking the covalently linked phosphates at the second and third serine residues. RR14 is the 14–amino acid functional domain derived from the parent Hist3 protein. The hybrid mixture candidates—DR9/2+RR14, DR9/2+Hist5, DR9/2+Hist3—are 50/50 physical mixtures (molarity based). The hybridized conjugates—DR9/2RR14, DR9/2Hist5, DR9/2Hist3—are the synthetically linked versions of the hybrid mixture candidates.

### Molecular Dynamics Simulations

All individual domains and hybridized conjugates were modeled by molecular dynamics (MD) simulations. Each polypeptide was prepared in a 12-Å TIP4PEW water box with physiologic concentrations of Na^+^ and Cl^–^ ions. The widely accepted AMBER14SB force field was used to describe the protein. Each system was minimized, heated, and equilibrated over multiple stages before 1-μs production simulations were generated with GROMACS 2021.4 (Lindahl et al. 2021). Trajectory analysis was also carried out via GROMACS 2021. Specifically, clustering was performed on the polypeptide backbone atoms to obtain MD representative structures. Secondary structure assignment was done with the DSSP program (Touw et al. 2015). The 3D hydrophobic moment (HM) vectors were calculated for the DR9/2 and RR14 regions of all representative structures via the 3D-HM web server (Reißer et al. 2014).

### Remaining Bioburden Analysis (Attached Biomass Assay)

The antibiofilm efficacy of protein/peptide candidates was tested against the *Streptococcus mutans* U159 as a model that is naturally competent and cariogenic with superior capability to metabolize a variety of carbohydrates (Burne 1998). A modified crystal violet assay was performed to analyze the biomass of 96-h biofilms of the exclusively attached bacterial cells to pellicle-coated hydroxyapatite (HAP) discs.

### Circular Dichroism Spectroscopic Analysis

Circular dichroism (CD) spectroscopy was used to measure the secondary structures of candidates suspended in the membrane-mimetic environment (Zhang et al. 2016). Estimations for secondary structures were obtained with BeStSel software (http://bestsel.elte.hu/index.php).

### Immobilization Capacity Analysis (Multiphoton Advanced Bioimaging)

Multiphoton microscopy was used to quantify the amount of protein/peptide molecules immobilized on human enamel in *ex vivo* mimicking conditions to identify their binding capacity. A total of 35 sound extracted human third molars were selected from a pool of unidentified extracted teeth obtained as surgical waste (exempt from institutional review board review) from the University of Saskatchewan dental clinics. After slicing, z-stacks were acquired for pellicle-coated enamel specimens with fluorescently labeled proteins/peptides via high–spatial resolution multiphoton microscopy (Ultima IV; Prairie Technology Inc.).

### Structure-Function Correlation

The correlation was calculated between the quantified immobilization capacity and the remaining bioburden on pellicle coatings of individual domains and their hybridized conjugates. The structure-functional relationship was displayed by plotting these 2 variables in relation to the MD simulations in the electrostatic surface display.

### Statistical Analysis

Statistical analyses were performed with the R statistical interface (version 4.0.2; R Core Team 2020). Analysis of variance and Tukey (honestly significant difference) post hoc tests were applied for multiple comparisons; *P* < 0.05 was considered statistically significant.

## Results

### MD Simulations

Each of the 4 individual domains and their 3 hybridized conjugates were modeled for 1 μs. The folding of the structures was heavily dependent on the hydrophobic interactions of the peptides, with certain amino acids being more hydrophilic (e.g., Arg, Asp, Lys) as compared with others that are highly hydrophobic (e.g., Phe, Leu, Tyr). Consequently, the surface of the molecule exposed to the surrounding water was generally hydrophilic in nature, as seen by the greater presence of yellow than white (Figure 1). Notably, the high hydrophilic region of DR9/2 (Glu-Glu-Lys) that is surrounded by hydrophobic residues may promote the formation of an α-helix in this region.

For each system, the folding of the polypeptide backbone was recorded over the trajectories (Appendix Table 1). Overall, all systems had relatively similar degrees of the 3-helix (4% to 10%), bend (9% to 19%), and turn (13% to 23%) structures and a significant portion of random coil. Except for Hist5, the amount of α-helix present was generally higher for the conjugated systems (up to 44%) than the individual domains (up to 12%).

The HM was determined for the DR9/2 and RR14 regions of the individual and conjugated systems. For the individual domains, DR9/2 had a larger HM vector length than RR14. Meanwhile, the HM of the RR14 was larger than the isolated domain when the region is incorporated into a longer sequence (Table). Likewise, this effect was greater for the conjugated polypeptides than for Hist3 and Hist5. The DR9/2Hist3 system had the longest HM vectors of all systems for the DR9/2 and RR14 regions (Table and Figure 1).

### Remaining Bioburden Analysis

**Figure 2.**
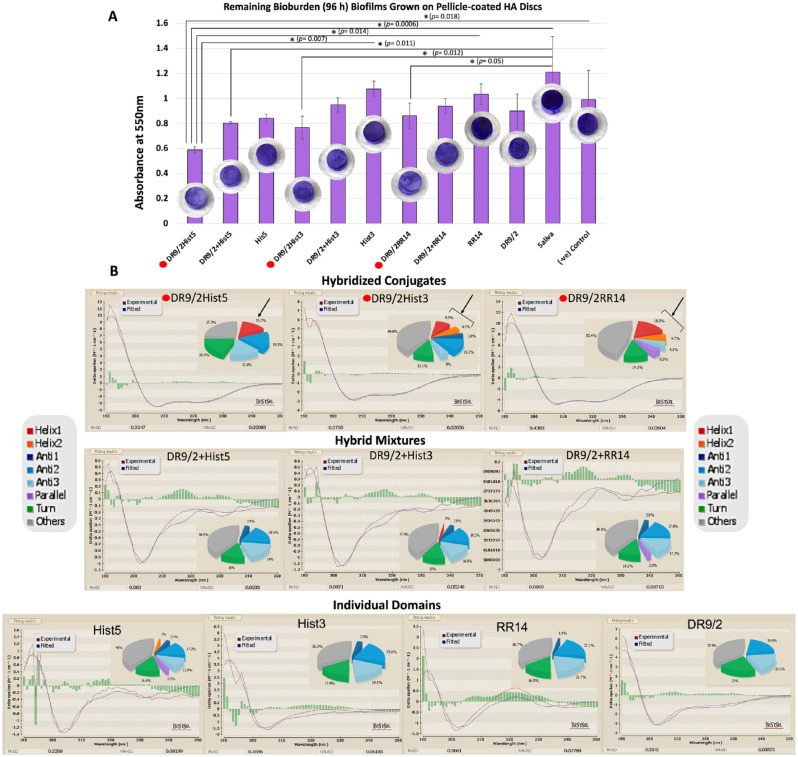
Structure-function characterization of acquired enamel pellicle–derived protein/peptide candidates, their hybrid mixtures, and their hybridized conjugates for biofilm dispersal. (**A**) Bioburden quantification for crystal violet–stained 96-h biofilms of *Streptococcus mutans* U159 grown on pellicle-coated hydroxyapatite (HAP) discs with protein/peptide candidates. All biofilms were grown on solid agar from exclusively attached cells to pellicle-coated HAP discs. Filtered pooled whole saliva was used as a positive control, while protein/peptide-free suspension buffer (0.01% acetic acid) was used as a negative control. Representative crystal violet–stained HAP discs (2 mm thick × 5 mm wide) for each group are depicted on the bar graphs. Asterisks show significant differences among groups: analysis of variance and Tukey honestly significant difference post hoc tests, *P* < 0.05. Mean ± SD. The red dots indicate the hybridized conjugates. (**B**) Molecular structural conformations of tested protein/peptide candidates show the circular dichroism spectra. Pie charts show estimation of secondary structure contents: α-helix (helix 1, regular; helix 2, distorted), β-sheets (antiparallel 1, left twisted; antiparallel 2, relaxed; antiparallel 3, right twisted; parallel), β-turn, and others (random coil). The black arrows denote the α-helix content. The red dots indicate the hybridized conjugates: these exclusively adopted an α-helix structure and showed the least bioburden. NRMSD, normalized root mean square deviation; RMSD, root mean square deviation.

For 96-h biofilms, all pellicles of the hybridized conjugates consistently grew less bioburden as compared with the hybrid mix or individual domains. All hybridized conjugates and DR9/2+Hist5 showed statistically significant differences in comparison with whole saliva, which recorded the highest bioburden. DR9/2Hist5 displayed the least bioburden and showed significant differences to the individual domains Hist3 and RR14 as well as the negative control. All *P* values are depicted in Figure 2A.

### CD Spectroscopic Analysis

All individual domains, except Hist5, and their hybrid mixtures did not adopt α-helical conformation; Hist5 showed 1% of distorted helix (Helix2) (Figure 2B). All candidates recorded a high content of random coil (38% to 48%). Upon conjugation, the hybridized conjugates showed a distinguished rise in the content of α-helical conformation: 15.2%, 14%, and 23% for DR9/2Hist5, DR9/2Hist3, and DR9/2RR14, respectively. Other measured secondary structures did not show any consistent pattern of change.

### Immobilization Capacity Analysis

**Figure 3.**
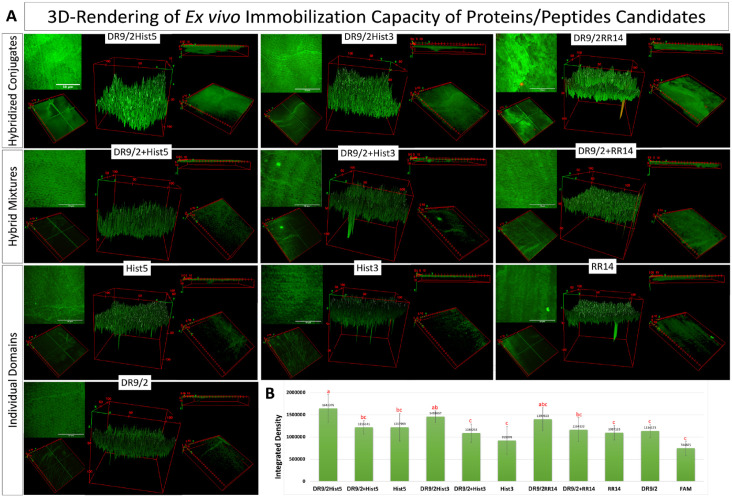
*Ex vivo* immobilization capacity of fluorescently labeled acquired enamel pellicle–derived proteins/peptides, their hybrid mixtures, and their hybridized conjugates on human enamel. (**A**) Multiphoton z-projections and 3-dimensional (3D) renderings of the immobilized protein/peptide candidates; 5 displays per candidate grouped in a cluster. Each cluster demonstrates the maximum intensity z-projection showing the highest attenuation value throughout the volume onto a 2D image (the scale bar is 50 μm) (top left). The 3D plot of the 2D image where the z-coordinate represents the brightness of each pixel (middle). The ortho middle slice of the z-stack shows the immobilization pattern halfway across the pellicle (bottom left). The 3D volume of the immobilized pellicle (bottom right) and its thickness (top right). (**B**) Quantification of the immobilization capacity (integrated density) of the tested candidates on human enamel. The FAM tag is the fluorescent molecule used to label proteins/peptides. Distinct lowercase letters show significant differences among groups: analysis of variance and Tukey honestly significant difference post hoc tests, *P* < 0.05. Mean ± SD.

Immobilized fluorescent protein/peptide molecules were quantified by calibrating the emitted fluorescence signals (Buijs and Hlady 1997). The mean integrated density was calculated by summing all pixels along the acquired z-stacks. Hybridized conjugates consistently showed significantly higher integrated density as compared with the hybrid mixtures and individual domains. DR9/2Hist5 recorded the highest integrated density (immobilization capacity) while Hist3 recorded the lowest one. Signals were emitted from the tagging molecule solely for the FAM group. The statistical significances were depicted in Figure 3.

### Structure-Function Correlation

**Figure 4.**
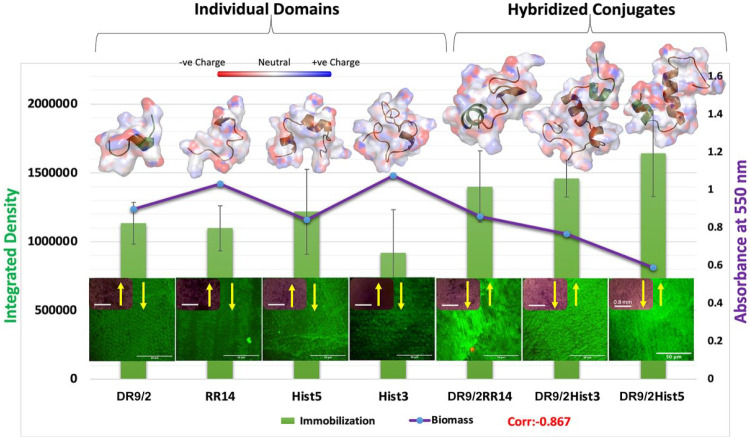
Structure-function relationship shows the correlation between the immobilization capacity of individual/conjugated protein/peptide candidates and the biomass formed on their pellicle coatings in relation to the adopted structural conformations. The primary y-axis shows the quantified immobilization capacity (integrated density) of individual/conjugated tested candidates presented in the green bar graph. The secondary y-axis shows the quantified 96-h *Streptococcus mutans* U159 biofilms grown on pellicle coatings of same candidates, presented in the purple line graph. A representative multiphoton z-projection image of the immobilization capacity is depicted for each candidate on the bottom, with an inset for inverted brightfield microscope characterization of the crystal violet–stained biofilm. On top, molecular dynamics simulations of the protein/peptide candidates are presented as an electrostatic surface display showing the adopted folded conformations. The negatively charged surfaces/atoms are shown in red and the positively charged are shown in blue.

The immobilization capacity displayed a negative correlation to the remaining bioburden (correlation = −0.867). The 2 negatively correlated phenotypes showed a relationship to the folding properties, whereas the hybridized conjugates—which adopt more folded conformations, as depicted in the MD simulations (Figure 4) and quantified in CD analysis (Figure 2B)—presented higher immobilization capacity and lower bioburden as compared with their individual domains.

## Discussion

Despite signiﬁcant health care advances and increased understanding of dental caries courtesy of unprecedented omics technologies, the etiology of dental caries remains poorly understood. Factually, untreated dental caries is still ranked number 1 among hundreds of assessed health-related conditions (Global Burden of Disease Collaborative Network 2020). Thus, there is a pressing need for preventative strategies to control this persistent health condition with in-depth mechanistic studies of caries pathogenesis aiming to the ultimate promise of personalized dentistry (Moussa, Ahmad, et al. 2022; Moussa, Sharma, et al. 2022).

In previous studies, we developed bioinspired multifunctional enamel pellicles embracing a mixture of synergically acting protein/peptide functional domains for efficient biofilm dispersal (Moussa and Siqueira 2021). Intriguingly, covalent conjugation of these domains induced a remarkable boosted and sustainable biofilm suppression effect for several days, showing that conjugation resulted in greater effects than simply the combined individual effects of the physical mixture of the constituent domains (Moussa and Siqueira 2022). In fact, the expressed antibiofilm behavior of proteins/peptides originates from a combination of physicochemical and structural features: size, residue composition, overall charge, secondary structure, hydrophobicity, and amphipathicity (Fjell et al. 2011). Accordingly, this study investigated the physicochemical properties of our *de novo* hybridized conjugates and simulated their 3D structural evolution (Table, Figure 1) to identify key features underlying their enhanced bioactivity.

The shared domain DR9/2, derived from the parent protein statherin and mutually conjugated to the histatins-derived domains, was crucial in leveraging several physicochemical characteristics and thus the subsequent folding and biological functions. On one hand, DR9/2 had the highest aliphatic index (indicator for thermal stability) and lowest GRAVY index (hydropathy) as compared with the other tested candidates, which notably improved resultant thermal stability and hydrophobicity of the hybridized conjugates (Table). On the other, DR9/2 negative charge, which is a limiting factor for the electrostatic interaction with bacteria, is reversed upon conjugation with the high positively charged histatin proteins/domains. This copresence of hydrophobic and polar (charged) residues within the conjugates generated strong amphipathic features and triggered the adoption of structural helical conformations that are central to the expressed bioactive properties (Kim et al. 2018); (Figure 1).

Visualizing these structural changes under simulative low-pH cariogenic conditions was enabled via MD simulations, which provide information about the dynamics and folding of the protein/peptide by calculating forces between atoms, including intramolecular hydrogen bonding and solvent interactions (Figure 1). Additionally, the calculated HM vector, which serves as a useful descriptor of macromolecular shape-dependent properties, was based on conformational ensembles from MD simulations. This 3D HM vector extended the classical 2D HM concept of measuring helix amphipathicity (Eisenberg et al. 1982) by accounting for all conformational rearrangements within a whole molecule (e.g., partial unfolding, bending, side-chain torsion angles) (Reißer et al. 2014). Typically, HM vectors increase in length with more unbalanced distribution of polar and nonpolar surface areas, whereas symmetrical arrangements of charges cancel the HM (Reißeret al. 2014). For the individual domains, DR9/2 had a larger HM vector length than RR14 (Table); this could be attributed to the unbalanced polarity of the former, given the high central hydrophilic region of DR9/2 neighboring strong hydrophobic residues (Figure 1). Furthermore, the considerable increase in the HM vector length for the RR14 region of the hybridized conjugates (Table) infers the emergence of a very unequal polarity distribution along the helix when the RR14 region is incorporated into a longer sequence. Particularly, this longer sequence includes the common conjugated DR9/2 region with its characteristic charge/polarity features as mentioned earlier. However, since simplifications have to be made in computational approaches, the methods derived from an empirical force field is inevitably limited by the complexity of natural systems (Fjell et al. 2011). Also, further comparison of simulations in solution versus in lipid membranes are still required since the peptides undergo instantaneous internal conformational rearrangement upon interaction with bacteria.

Nonetheless, our CD analysis experimentally validated the MD predictions for structural amphipathicity; the hybridized conjugates exclusively showed considerable content of α-helical conformation in the membrane-mimetic environment (Figure 2). Plus, the observed coherent association between the adopted α-helices and antibiofilm potency was evident where hybridized conjugates grew consistently less biofilm as compared with their individual domains or hybrid mixes.

The efficacious role of α-helices in biofilm suppression has been well established in the literature not only for biofilm dispersal but also for controlling the volume, thickness, and channel formation of biofilms (Guilhen et al. 2017). Although the precise details of the mechanism of action of α-helices are not fully unraveled, their amphipathicity (surfactant-like features) is key in dispersing biofilms. Likewise, several other amphipathic molecules have been proposed to promote biofilm dispersal (Guilhen et al. 2017). For designing antimicrobial peptides, incorporating cationic amino acids to an overall positive charge (+2 to +9) and a large percentage (≥30%) of hydrophobic amino acids is a prerequisite for permitting peptides to fold into amphipathic conformations (Zhang et al. 2016; Kim et al. 2018). Moreover, some studies noted the significance of coexistence of positively charged lysine and hydrophobic leucine for strong helix formation and antimicrobial potency through amplifying the hydrophobicity within the amphipathic structure (Blondelle and Houghten 1992; Beven et al. 2003). Taken together, the strong and sustainable biofilm suppression expressed by our designed conjugates could be explained by their adoption of well-stabilized α-helix structure, given that these conjugates have fulfilled all the aforementioned key folding requisites. Still, it is difficult to estimate the exact contributions of the previously stated physicochemical and structural parameters to the conveyed bioactivity; hence, these parameters are not necessarily independent (Zhang et al. 2016). Therefore, further structural-functional investigations are needed to define more specific correlations, an optimal range of structural parameters, and parameter balancing.

Aiming for translational potential, we investigated the immobilization/binding capacity of these designed pellicles on enamel tissue using multiphoton high–spatial resolution microscopy. It has a distinguished capability for optical sectioning by suppressing background signals, optimizing signal-to-noise ratio, and reducing phototoxicity to the focal region by employing the noninvasive near-infrared femtosecond lasers (Larson 2011; Lefort 2017). Our expertise in developing multiphoton-based imaging protocols specifically tailored for biological hard dental tissue (Moussa, Kirihara, et al. 2019; Moussa and Aparicio 2020; Moussa and Siqueira 2021) enabled a thorough *ex vivo* optical sectioning analysis of pellicle-coated human enamel (Figure 3).

The significantly increased immobilization capacity of the conjugates, as compared with the individual domains and hybrid mixtures, triggered our curiosity (Figure 3). The biofilm dispersal effect of α-helices has been well studied, as detailed earlier; however, we questioned how this adopted conformation enhances binding capacity and which other factors make these conjugates strongly immobilized. Barely a handful of protein structures in biomineral systems have been characterized in detail (Shaw et al. 2020). Relevantly, Long et al. (2001) used solid-state nuclear magnetic resonance spectroscopy to provide high-resolution structural and dynamic characterization of salivary statherin adsorbed to HAP surface. They revealed that the N-terminus residues directly bound to HAP in hydrated conditions and characteristically adopted an α-helical character. They added that the binding footprint to HAP is mostly confined to the highly anionic and acidic residues at the N-terminal pentapeptide region (aspartic and glutamic acids), besides the region’s stabilized α-helical conformation (Long et al. 2001). Likewise, others have proposed α-helix as a general structural mechanism for aligning acidic side chain residues with HAP and have highlighted the statherin N-terminal pentapeptide sequence as a common motif in several bone- and tooth-immobilized proteins (Raj et al. 1992). Furthermore, in a data-mining study for the salivary and pellicle proteome, the authors found that pellicle-bound proteins show more helical structures as compared with saliva proteins (Schweigel et al. 2016). Collectively, these findings plausibly explain the increased immobilization of our conjugates, which not only adopted high helical content but also encompassed the N-terminus 9–amino acid residues of statherin as a mutual motif with other histatin-derived domains (Figure 4).

Still, the helical conformation that we identified was in a solution state, a limitation of CD characterization, while proteins mostly undergo large conformational changes upon adsorption to solid surfaces (Shaw et al. 2020). Therefore, further studies with solid-state nuclear magnetic resonance spectroscopy and/or *in situ* atomic force microscopy are warranted to reveal the *in situ* folding and kinetics of our conjugates upon HAP or enamel binding.

Plotting the biomass grown on our individual/conjugated protein/peptide pellicle coatings and their immobilization capacity on enamel revealed a strong negative correlation, where both phenotypes were primarily linked to α-helix structural stabilization (Figures 2, 4); (Long et al. 2001; Fjell et al. 2011). Accordingly, we may conclude that the stabilized helical confirmation is regarded as a mechanistic controller that underlies pellicle binding and thus the consequent expression of bioactivity. Yet, this presented bioactivity was in respect to a single-species model. So, further studies in a saliva- or biofilm-derived multispecies environment are required for additional validation.

## Conclusions

Integrating complementary-acting bioactive proteins/peptides is a contemporary translational approach to develop efficacious preventative agents for caries and other dental diseases. Our *de novo* antibiofilm protein/peptide hybridized conjugates, engineered to pellicle-coated enamel, showed a relation among high α-helix content, immobilization capacity on human enamel, and sustained biofilm suppression for several days. Embracing certain physicochemical properties of the constituent domains that trigger stabilized helical conformations is key for boosting bioactivity and immobilization capacity on dental hard tissues. These data can guide optimal selections for bioengineering versatile therapeutics to prevent/arrest dental caries, which otherwise requires invasive treatments with significant costs.

## Author Contributions

D.G. Moussa, contributed to conception and design, data acquisition, analysis, or interpretation, drafted and critically revised the manuscript; R.W. Kung, contributed to data acquisition, analysis, or interpretation, drafted the molecular dynamics simulations and critically revised the manuscript; J.S. Tse, contributed to data conception and design, critically revised the manuscript; W.L. Siqueira, contributed to data conception and design, drafted and critically revised the manuscript. All authors gave their final approval and agree to be accountable for all aspects of the work.

## Supplemental Material

sj-docx-1-jdr-10.1177_00220345231162336 – Supplemental material for Mechanistic Insights into Bioengineered Antibiofilm Enamel Pellicles Supplemental material, sj-docx-1-jdr-10.1177_00220345231162336 for Mechanistic Insights into Bioengineered Antibiofilm Enamel Pellicles by D.G. Moussa, R.W. Kung, J.S. Tse and W.L. Siqueira in Journal of Dental Research
