# Supplementary material for: Mechanistic Insights into Bioengineered Antibiofilm Enamel Pellicles
Source: J Dent Res. 2023 Apr 21;102(7):743–51. doi: 10.1177/00220345231162336 (PMC10288165; doi:10.1177/00220345231162336)
Supplement: sj-docx-1-jdr-10.1177_00220345231162336 – Supplemental material for Mechanistic Insights into Bioengineered Antibiofilm Enamel Pellicles [file sj-docx-1-jdr-10.1177_00220345231162336.docx]

**Structure-Function Insights into Bioengineered Antibiofilm Enamel Pellicle Proteins**

**AUTHORS: Dina G. Moussa^1^, Ryan W. Kung^2^, John S. Tse^2^, Walter L. Siqueira^1*^**

**^1^**College of Dentistry, University of Saskatchewan, Saskatoon, Saskatchewan, CANADA

^2^Department of Physics and Engineering Physics, College of Art and Science, University of Saskatchewan, Saskatoon, Saskatchewan, CANADA

*Correspondent Author:

Walter L. Siqueira DDS PhD FCAHS

Dean and Professor

Email: [walter.siqueira@usask.ca](mailto:walter.siqueira@usask.ca)

College of Dentistry, University of Saskatchewan

**Keywords:** Enamel Pellicle, Salivary Proteins and Peptides, Bio-Engineering, Molecular Dynamics Simulation, 3-D Imaging, Biofilm

**Appendix Materials and Methods**

***Human participants***

Participants included healthy, non-smoking adult male and female volunteers (25 to 40 years old) with no signs of periodontal diseases, active dental caries, or any other oral or systemic condition that could affect whole saliva composition. Saliva samples were freshly collected 2h after breakfast, between 9:00 am to 11:00 am, and then pooled to minimize the circadian rhythm differences and biological variations, respectively. Samples were collected in sterile 14-ml polypropylene tubes (Corning, NY, USA) chilled on ice, spun down, and filter-sterilized using 0.2 μm syringe filters.

***Molecular Dynamics (MD) Simulations.***

The individual protein/peptide domains and their hybridized conjugates were modelled using molecular dynamics (MD) simulations, namely (DR9/2, RR14, Histatin5 (Hist5), Histatin3 (Hist3), DR9/2RR14, DR9/2Hist5 and DR9/2Hist3). Robetta ([Baek et al. 2021](#_ENREF_2)) was used for the structural prediction of the DR9/2Hist5, DR9/2Hist3 and Hist3 peptides due to the 26 minimum number of AAs required to generate structures. Consequently, the DR9/2, RR14 and Hist5 monomeric units were then obtained by extracting the residues of the small polypeptides from the previously generated structures. Lastly, an initial structure of DR9/2RR14 was generated by overlaying the protein backbone of RR14 onto the Hist3 section of DR9/2Hist3 and extracting the desired residues.

PlayMolecule ProteinPrepare ([Martínez-Rosell et al. 2017](#_ENREF_8)) was used to assign protonation states to each polypeptide based on a pH of 5. Each generated polypeptide structure was prepared for MD using the leap module of Amber20 ([Case et al. 2020](#_ENREF_3)) to neutralize the system and add physiological concentrations of Na+ and Cl- ions. Subsequently, a TIP4PEW water box was added such that there was at least 12 Å between the edge of the box and the solute. The widely accepted AMBER14SB ([Maier et al. 2015](#_ENREF_7)) forcefield was used to describe the protein.

The systems were minimized over four stages with Amber20 ([Case et al. 2020](#_ENREF_3)), with each stage consisting of 1000 steps using the steepest decent minimization algorithm followed by 3000 steps using conjugate gradient algorithm. A 100 kcal/(mol Å^2^) force restraint was applied to the solute in stage 1, solvent and hydrogens in stage 2, and solvent in stage 3, with the last minimization stage being done without restraints. Each system was then heated by increasing the temperature to 300 K in six 20 ps stages at constant volume, with a 25 kcal/(mol Å^2^) restraint applied to the solute. Next, equilibration was then performed for 100 ps, with the restraint on the solute weakened every 20 ps.

Production simulations were run for using GROMACS 2021.4 ([Abraham et al. 2015](#_ENREF_1); [Lindahl et al. 2021](#_ENREF_6)) for 1 μs. During the production phase, LINCS ([Hess et al. 1997](#_ENREF_4)) holonomic constraints were used with a 2 fs timestep. A nonbonded cutoff of 10 Å was implemented for all simulations along with the periodic boundary condition.

Trajectory analysis was carried out using GROMACS 2021.4 ([Abraham et al. 2015](#_ENREF_1); [Lindahl et al. 2021](#_ENREF_6)). Specifically, clustering was performed on the polypeptide backbone atoms using a single linkage algorithm to obtain MD representative structures. Secondary structure assignment was done using the DSSP program ([Kabsch and Sander 1983](#_ENREF_5); [Touw et al. 2015](#_ENREF_14)). The 3D Hydrophobic Moment (HM) Vectors were calculated for the DR9/2 and RR14 regions of all seven representative structures using the 3D-HM webserver ([Reißer et al. 2014](#_ENREF_11)). Specifically, the HM was determined for the DR9/2 and/or the RR14 region for each system.

***Remaining bioburden analysis (attached biomass assay).***

Planktonic bacteria was grown aerobically (10% CO2) in brain heart infusion (BHI) medium with 1% sucrose supplement (BD BBL™ #211059, MD, USA) overnight. Total of 36 Hydroxyapatite (HAP) discs, 5mm wide and 2 mm thick, were marked at one side, autoclaved, and used as substrates to be pellicle-coated with protein/peptide tested candidates before growing biofilms. Each HAP disc, the unmarked surface facing up, was pellicle-coated with 100 μl of 200 μM of protein/peptide candidates or controls for 2 h at 37 °C under gentle shaking. Afterwards, the unbound proteins/peptides were passively aspirated and 100 μl the adjusted inoculum, (5.0×106 CFU/ml) in Phosphate Buffered Saline (PBS), was dispensed. The inoculated HAP discs were incubated for 3-4 h under same conditions mentioned above to promote the attachment of bacterial cells to pellicle-coated HAP discs. Then the discs were PBS-rinsed 2-3 times, let to dry for 15 min, and loaded upside-down on agar plates, where the inoculated surfaces facing the agar. Agar plates were incubated for 96 hours then the HAP discs were crystal violet (0.1%) stained to quantify the biofilms of the exclusively attached bacterial cells to pellicle coatings. All formed biofilms were characterized with a bright field inverted microscope (EVOS™ xl core, MA, USA) before dissolving the satin, with 33% acetic acid, and reading at OD 550 nm. The assay was conducted 3 independent times with sample size n = 5.

***Circular dichroism (CD) spectroscopic analysis.***

The circular dichroism analysis was performed to determine the secondary structures and folding properties of tested candidates. The experiments were performed using the Chirascan-plus CD spectrometer (Applied Photophysics), at the Protein Characterization and Crystallization Facility (PCCF), University of Saskatchewan. All experiments were performed in a quartz cuvette with a path length of 0.1 mm (Hellma, Germany). 50% Trifluoroethanol (TFE) was used as membrane-mimetic environment ([Takahashi et al. 2010](#_ENREF_13); [Zhang et al. 2016](#_ENREF_15)) and was calibrated in comparison to the standard PBS buffer to collect a baseline measurement at the wavelengths 190-260 nm. The concentration of each sample was adjusted individually to obtain good signals determined by the total absorbance level. The smaller peptides required higher concentrations than the larger ones. The least concentrations used was 50 uM and the highest was 200uM. Data was collected in triplicate and the average CD spectrum for each sample was obtained after a baseline correction was performed. Estimations for the different secondary structure contents were obtained from further analysis of CD spectra using BeStSel (Beta Structure Selection) method (<http://bestsel.elte.hu/index.php>) as described in ([Micsonai et al. 2015](#_ENREF_9)) .

***Immobilization capacity analysis (multiphoton advanced bioimaging).***

A total of 35 sound extracted human third molars were selected from a pool of unidentified extracted teeth, that is exempt from IRB review, previously obtained as surgical waste from the dental clinics at the University of Saskatchewan~~.~~ The roots were sectioned using a diamond saw (IsometTM, Buehler, Lake Bluff, IL, USA) and discarded. Then, coronal proximal slices were sectioned occluso-cervically from the mesial and distal sides of each tooth. The sliced specimens were then ground to 0.5 mm, polished with 320-, 600-, and 800-grit Si-C papers using a polishing machine (Ecomet 3, Buehler, IL, USA), and ultrasonicated in a water bath for 20 min ([Moussa et al. 2019](#_ENREF_10)). Pellicle coatings were obtained by incubating the enamel specimens with 200 ul of 200 uM fluorescently labelled proteins/peptides for 2 h under gentle shaking in dark condition. Afterwards, specimens were rinsed three times with distilled water and kept on ice for imaging. Z-stacks of pellicle-coated enamel slices were acquired with the multiphoton microscope (Ultima IV, Prairie Technology Inc., WI, USA). Non-descanned high sensitivity multialckali photocatode photomultiplier detectors (R3896, Hamamatsu, Japan) were used to allow visualization of multiple fluorophores located deep within the specimen. The infra-red (IR) laser (MAI TAI, Spectra-Physics, USA), tuned at 870 nm, was operated in pulsed mode using a 40 × NA 0.8 water immersion objective (LUMPlanFl/IR, Olympus, Japan) with a 2 mm working distance. Solutions of the tagging molecule (FAM) was used to pellicle-coat the control specimens. The samples were scanned in 512x512 pixel frames and 50 µm of enamel thickness was sampled with a 3 µm step size in Z. Scanning was conducted 3 independent times, using slices from 3 independent teeth, with sample size n = 3/each. Images were compiled and analyzed with FIJI software (Fiji Is Just) ImageJ, version: 2.1.0/1.53c <https://imagej.net/Contributors> ([Schindelin et al. 2012](#_ENREF_12)).

**Appendix Results**

**Appendix Table 1.** The relative frequency of protein secondary structures over the 1μs molecular dynamics trajectories for each protein/peptide modelled, with the ratios of each conformation being dependent on the exact amino acid sequence. Structures from the simulations were analyzed every 100 ps using the DSSP program within GROMACS 2021.4, which determines the secondary structure based on the backbone hydrogen bonding.

|  | **Coil** | **B-Sheet** |  | **B-Bridge** | **Bend** | **Turn** | **A-Helix** | **5-Helix** | **3-Helix** |
| --- | --- | --- | --- | --- | --- | --- | --- | --- | --- |
| **DR9/2** | 0.51 | 0 |  | 0 | 0.12 | 0.23 | 0.05 | 0 | 0.1 |
| **RR14** | 0.61 | 0.01 |  | 0.01 | 0.18 | 0.13 | 0.03 | 0 | 0.04 |
| **Hist5** | 0.31 | 0 |  | 0.01 | 0.12 | 0.16 | 0.33 | 0 | 0.07 |
| **Hist3** | 0.38 | 0 |  | 0 | 0.19 | 0.2 | 0.12 | 0.04 | 0.07 |
| **DR9/2RR14** | 0.36 | 0.01 |  | 0 | 0.16 | 0.22 | 0.19 | 0 | 0.06 |
| **DR9/2Hist5** | 0.38 | 0 |  | 0 | 0.19 | 0.2 | 0.12 | 0.04 | 0.07 |
| **DR9/2Hist3** | 0.21 | 0 |  | 0.01 | 0.09 | 0.18 | 0.44 | 0.01 | 0.06 |

**References:**

Abraham MJ, Murtola T, Schulz R, Páll S, Smith JC, Hess B, Lindahl E. 2015. Gromacs: High performance molecular simulations through multi-level parallelism from laptops to supercomputers. SoftwareX. 1-2:19-25.

Baek M, DiMaio F, Anishchenko I, Dauparas J, Ovchinnikov S, Lee GR, Wang J, Cong Q, Kinch LN, Schaeffer RD et al. 2021. Accurate prediction of protein structures and interactions using a three-track neural network. Science. 373(6557):871-876.

Case DA, Belfon K, Ben-Shalom IY, Brozell SR, Cerutti DS, T.E. Cheatham I, Cruzeiro VWD, Darden TA, R.E. Duke GG, M.K. Gilson, H. Gohlke, A.W. Goetz,R Harris, S. Izadi, S.A. Izmailov, K. Kasavajhala, A. Kovalenko, R. Krasny, T. Kurtzman, T.S. Lee, S. LeGrand, P. Li, C. Lin, J. Liu, T. Luchko, R. Luo, V. Man, K.M. Merz, Y. Miao, O. Mikhailovskii, G. Monard, H. Nguyen, A. Onufriev, F., Pan SP, R. Qi, D.R. Roe, A. Roitberg, C. Sagui, S. Schott-Verdugo, J. Shen, C.L. Simmerling, N.R. Skrynnikov, J. Smith, J. Swails, R.C. Walker, J. Wang, L. Wilson, R.M. Wolf, X. Wu, Y. Xiong, Y. Xue, D.M. York and P.A. Kollman. 2020. Amber 2020. San Francisco: University of California.

Hess B, Bekker H, Berendsen HJC, Fraaije JGEM. 1997. Lincs: A linear constraint solver for molecular simulations. Journal of Computational Chemistry. 18(12):1463-1472.

Kabsch W, Sander C. 1983. Dictionary of protein secondary structure: Pattern recognition of hydrogen-bonded and geometrical features. Biopolymers. 22(12):2577-2637.

Lindahl, Abraham, Hess, Spoel vd. 2021. Gromacs 2021.4 source code. Zenodo.

Maier JA, Martinez C, Kasavajhala K, Wickstrom L, Hauser KE, Simmerling C. 2015. Ff14sb: Improving the accuracy of protein side chain and backbone parameters from ff99sb. Journal of Chemical Theory and Computation. 11(8):3696-3713.

Martínez-Rosell G, Giorgino T, De Fabritiis G. 2017. Playmolecule proteinprepare: A web application for protein preparation for molecular dynamics simulations. Journal of Chemical Information and Modeling. 57(7):1511-1516.

Micsonai A, Wien F, Kernya L, Lee YH, Goto Y, Refregiers M, Kardos J. 2015. Accurate secondary structure prediction and fold recognition for circular dichroism spectroscopy. Proc Natl Acad Sci U S A. 112(24):E3095-3103.

Moussa DG, Kirihara JA, Ye Z, Fischer NG, Khot J, Witthuhn BA, Aparicio C. 2019. Dentin priming with amphipathic antimicrobial peptides. J Dent Res. 98(10):1112-1121.

Reißer S, Strandberg E, Steinbrecher T, Ulrich AS. 2014. 3d hydrophobic moment vectors as a tool to characterize the surface polarity of amphiphilic peptides. Biophys J. 106(11):2385-2394.

Schindelin J, Arganda-Carreras I, Frise E, Kaynig V, Longair M, Pietzsch T, Preibisch S, Rueden C, Saalfeld S, Schmid B et al. 2012. Fiji: An open-source platform for biological-image analysis. Nat Methods. 9(7):676-682.

Takahashi D, Shukla SK, Prakash O, Zhang G. 2010. Structural determinants of host defense peptides for antimicrobial activity and target cell selectivity. Biochimie. 92(9):1236-1241.

Touw WG, Baakman C, Black J, te Beek TA, Krieger E, Joosten RP, Vriend G. 2015. A series of pdb-related databanks for everyday needs. Nucleic Acids Res. 43(Database issue):D364-368.

Zhang SK, Song JW, Gong F, Li SB, Chang HY, Xie HM, Gao HW, Tan YX, Ji SP. 2016. Design of an alpha-helical antimicrobial peptide with improved cell-selective and potent anti-biofilm activity. Sci Rep. 6:27394.
